# Placental polyamine metabolism differs by fetal sex, fetal growth restriction, and preeclampsia

**DOI:** 10.1172/jci.insight.120723

**Published:** 2018-07-12

**Authors:** Sungsam Gong, Ulla Sovio, Irving L.M.H. Aye, Francesca Gaccioli, Justyna Dopierala, Michelle D. Johnson, Angela M. Wood, Emma Cook, Benjamin J. Jenkins, Albert Koulman, Robert A. Casero, Miguel Constância, D. Stephen Charnock-Jones, Gordon C.S. Smith

**Affiliations:** 1 Department of Obstetrics and Gynaecology, NIHR Cambridge Comprehensive Biomedical Research Centre,; 2 Centre for Trophoblast Research (CTR), Department of Physiology, Development and Neuroscience,; 3 Department of Public Health and Primary Care,; 4 NIHR BRC Core Metabolomics and Lipidomics Laboratory, University of Cambridge, Cambridge, United Kingdom.; 5 The Sidney Kimmel Comprehensive Cancer Center, Johns Hopkins University School of Medicine, Baltimore, Maryland, USA.; 6 University of Cambridge Metabolic Research Laboratories and MRC Metabolic Diseases Unit, Wellcome Trust-MRC Institute of Metabolic Science, Cambridge, United Kingdom.

**Keywords:** Reproductive Biology, Expression profiling, Obstetrics/gynecology, Polyamines

## Abstract

Preeclampsia and fetal growth restriction (FGR) are major causes of the more than 5 million perinatal and infant deaths occurring globally each year, and both are associated with placental dysfunction. The risk of perinatal and infant death is greater in males, but the mechanisms are unclear. We studied data and biological samples from the Pregnancy Outcome Prediction (POP) study, a prospective cohort study that followed 4,212 women having first pregnancies from their dating ultrasound scan through delivery. We tested the hypothesis that fetal sex would be associated with altered placental function using multiomic and targeted analyses. We found that spermine synthase (*SMS*) escapes X-chromosome inactivation (XCI) in the placenta and is expressed at lower levels in male primary trophoblast cells, and male cells were more sensitive to polyamine depletion. The spermine metabolite N1,N12-diacetylspermine (DiAcSpm) was higher in the female placenta and in the serum of women pregnant with a female fetus. Higher maternal serum levels of DiAcSpm increased the risk of preeclampsia but decreased the risk of FGR. To our knowledge, DiAcSpm is the first maternal biomarker to demonstrate opposite associations with preeclampsia and FGR, and this is the first evidence to implicate polyamine metabolism in sex-related differences in placentally related complications of human pregnancy.

## Abstract 2

The human female placenta has enhanced spermine synthesis which protects fetal growth but predisposes to preeclampsia.

## Graphical abstract

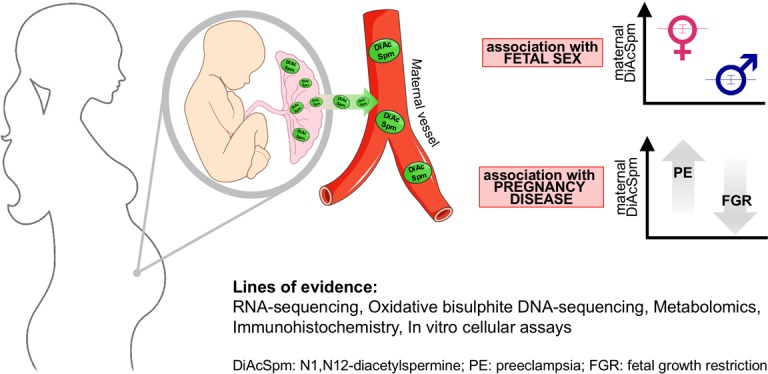

## Introduction

Preeclampsia and FGR are 2 of the “great obstetrical syndromes” (1). These disorders cause short-term complications for the infant and have been associated with a range of diseases in adult life, such as ischemic heart disease, stroke, type 2 diabetes, and long-term neurodevelopmental disorders (2). Moreover, these complications have profound effects on maternal morbidity and mortality. Preeclampsia is one of the leading causes of maternal death globally, and both preeclampsia and FGR are markers for the mother’s later risk of cardiovascular disease (3). Both preeclampsia and FGR are associated with abnormal placental function and metabolism (4). Currently, the placental mechanisms leading to preeclampsia and FGR are only partially understood. It is also unknown why placental dysfunction can lead to preeclampsia without FGR in one woman and FGR without preeclampsia in another. Epidemiological studies have demonstrated that fetal sex is associated with different patterns of ultrasonic markers of placentation (5), placental pathology (6), the risk of perinatal death (7–10), and the risk of preeclampsia (11, 12), but the mechanisms underlying these differences are unknown. Given these observations, we hypothesized that the placenta would exhibit sex-related differences across multiomic analyses. Moreover, we hypothesized that sex-related differences in placental function would be associated with the risk of placentally related complications of human pregnancy. We tested these hypotheses using 3 discovery-based methods, namely analysis of the placental transcriptome, the placental methylome, and the mother’s serum metabolome. We then used targeted in vitro experiments in primary cultures of human trophoblast cells to confirm sex-related differences in function.

## Results

### Sex-related transcriptome differences are driven by genes escaping XCI in the placenta.

**Figure 1:**
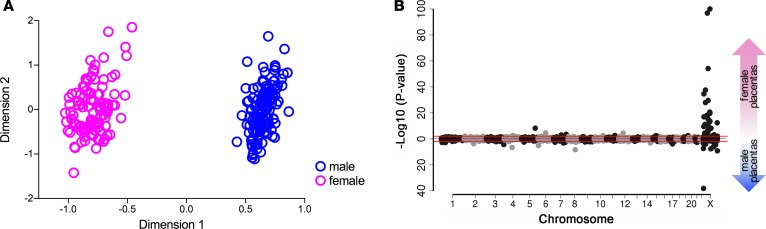
Analysis of the placental transcriptome by fetal sex. (**A**) Multidimensional scaling plot of RNA-seq data. (**B**) Manhattan plot of sex-biased genes on autosomes and X chromosome. *P* values on the *y* axis, Benjamini-Hochberg corrected (DESeq2). Red lines indicate adjusted *P* = 0.01.

**Figure 2:**
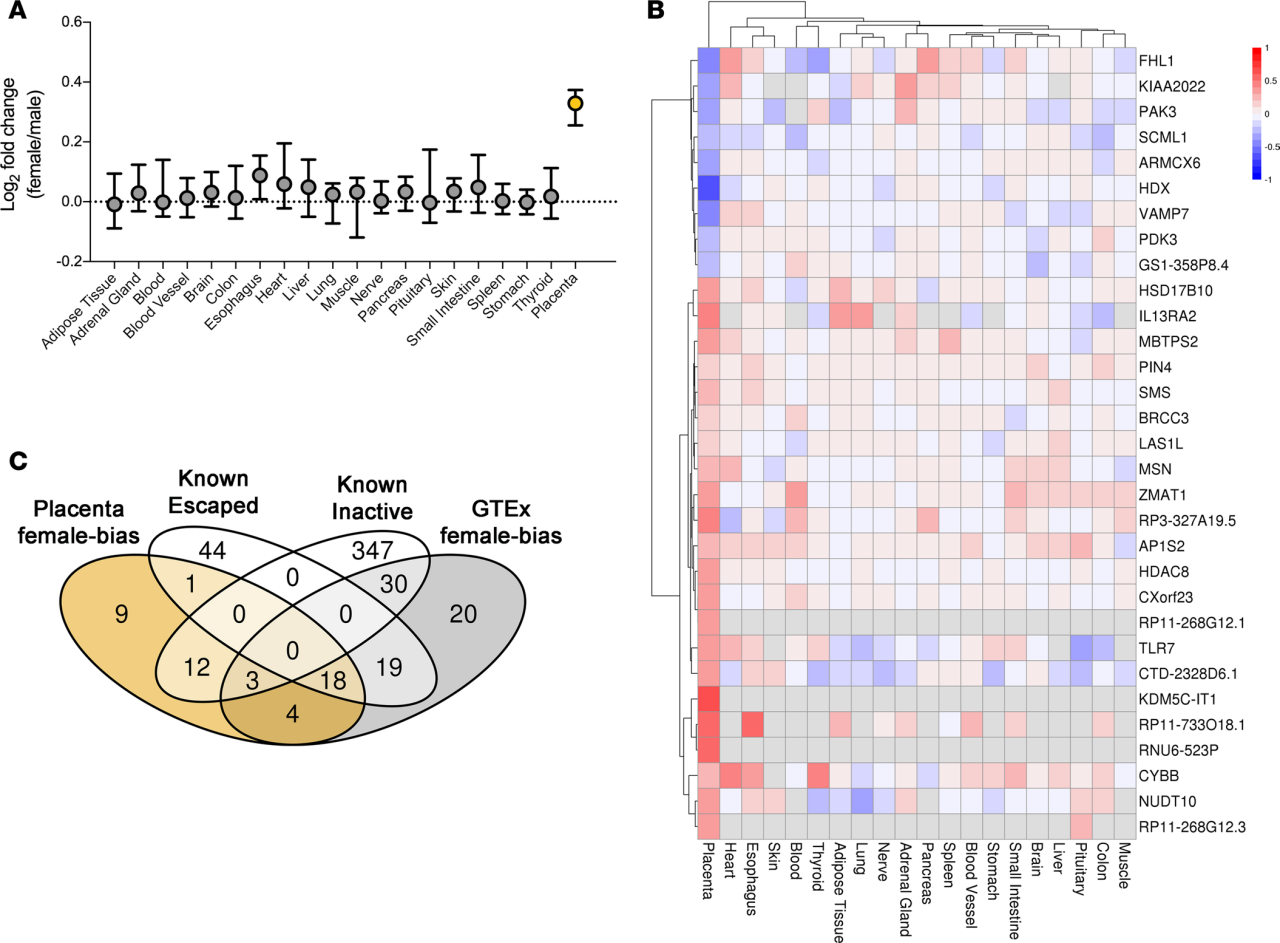
Placental enrichment of genes escaped from X-chromosome inactivation. (**A**) Transcript abundance differences in placenta and 19 other tissues (GTEx) of the 22 genes with placental female-biased expression. Points, median; bars, interquartile range (IQR). (**B**) Heatmap of expression (log_2_ fold change) of previously unrecognized sex-biased X chromosome genes in placenta and 19 GTEx tissues. Red, female-biased (*n* = 22); blue, male-biased (*n* = 9); gray, low expression (read-count < 10). (**C**) Venn diagram of overlap between X chromosome genes with female-biased expression in placenta (placenta, female-bias) and in the 19 GTEx tissues (GTEx, female-bias), and genes previously reported to escape (known escaped) or to be subjected (known inactive) to X-inactivation.

We recruited nulliparous women to a study that involved serial antenatal blood sampling, blinded ultrasound scans, and postpartum placental sampling and followed 4,212 participants from early pregnancy through birth (Table 1) (13). We compared the placental transcriptome profile of 67 male and 64 female healthy placentas using RNA sequencing (RNA-seq). We collected about 100 M single-end 125 base reads from each subject (Supplemental Table 1; supplemental material available online with this article; https://doi.org/10.1172/jci.insight.120723DS1). A multidimensional scaling plot demonstrated profound clustering of the placental transcriptome by fetal sex (Figure 1A). After exclusion of Y chromosome genes, we observed a highly significant enrichment of differentially expressed genes on the X chromosome over the autosomes (*P* = 1.8 × 10^–42^; Figure 1B and Supplemental Table 2). Using the previously described threshold of a 10% difference in male/female transcript abundance (plus a *P* < 0.01 threshold, see Methods for details) (14, 15), we identified 60 X chromosome genes with sex-biased expression in the placenta (Supplemental Table 3): 47 were female-biased and 13 were male-biased. We analyzed the publicly available RNA-seq dataset from 19 other human tissues from the Genotype-Tissue Expression (GTEx) project (16, 17). The average number of X chromosome genes with female-biased expression was 27 (range 12–37, Supplemental Table 3), and 22 of the X chromosome genes exhibiting female-biased expression in the placenta did not exhibit female-biased expression in any of these other tissues (Figure 2 and Supplemental Table 4) (18).

**Figure 3:**
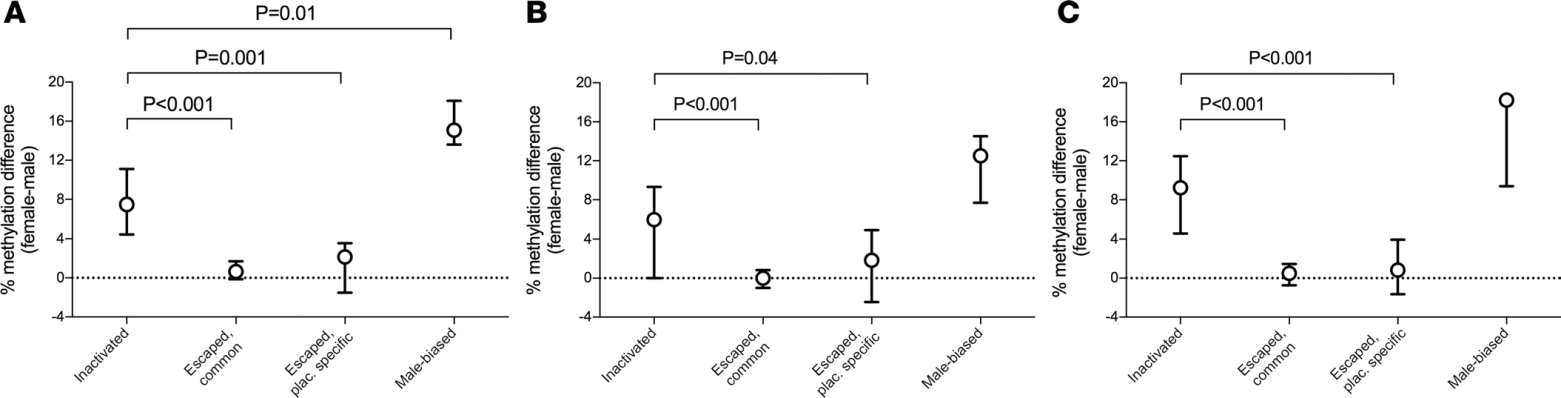
Promoter methylation analysis of X chromosome genes in male and female placentas. (**A**) Promoter methylation differences in male (*n* = 2) and female (*n* = 2) term placentas using WGoxBS profiles. (**B** and **C**) Technical and biological validation experiments performed using an in-solution methodology and bisufite conversion (SureSelectXT Methyl-Seq, Agilent) in (**B**) 2 placental samples (*n* = 1 male and *n* = 1 female) previously analyzed by WGoxBS and (**C**) a different set of male (*n* = 3) and female (*n* = 3) term placentas. The graphs show X chromosome genes subject to (inactivated) or escaped from X-chromosome inactivation, along with male-biased expression. Escaped genes uniquely identified in placenta (escaped, plac. specific) are drawn separately from those identified in at least one of 19 GTEx tissues (escaped, common). Points, median; bars, IQR. *P* values in the graphs: Mann-Whitney *U* test (each group vs. inactivated). Overall *P* = 0.0001 (**A**), *P* = 0.0007 (**B**), and *P* = 0.0001 (**C**) obtained with Kruskal-Wallis rank test.

Female-biased expression of X chromosome genes is commonly due to escape from X-chromosome inactivation (XCI) (19). Of the 47 female-biased genes in the placenta, only 19 had previously reported to escape from XCI (14, 15), and the other 28 were classified either as inactivated, variable, or unknown (Figure 2C and Supplemental Table 3). Unlike in the mouse, XCI in the human placenta is not parent-of-origin specific (20), and direct assessment of biallelic expression of X chromosome genes is not possible using analysis of mRNA from whole tissue biopsies. However, identifying genes that escape XCI is also informed by analysis of DNA methylation (19); escaped genes do not exhibit sex-related differences in promoter methylation, whereas the promoter regions of genes subject to XCI are generally hypermethylated in females. Therefore, we compared promoter methylation in healthy male and female placental samples using whole genome oxidative bisulphite DNA-seq (Figure 3A). Both the known and potentially novel placenta-specific escaped genes had similar levels of promoter methylation in male and female tissues, whereas genes without sex-biased expression demonstrated promoter hypermethylation in females. An analogous pattern was also observed in male-biased X chromosome genes, which exhibited promoter hypermethylation in females. We replicated these results in a further group of male and female samples using SureSelectXT Methyl-Seq (Agilent) (Figure 3, B and C, and Supplemental Tables 5 and 6). These findings indicated that the placenta had a unique profile of genes escaping XCI compared with other human tissues.

### Maternal serum levels of DiAcSpm differ by fetal sex and pregnancy complications.

**Figure 4:**
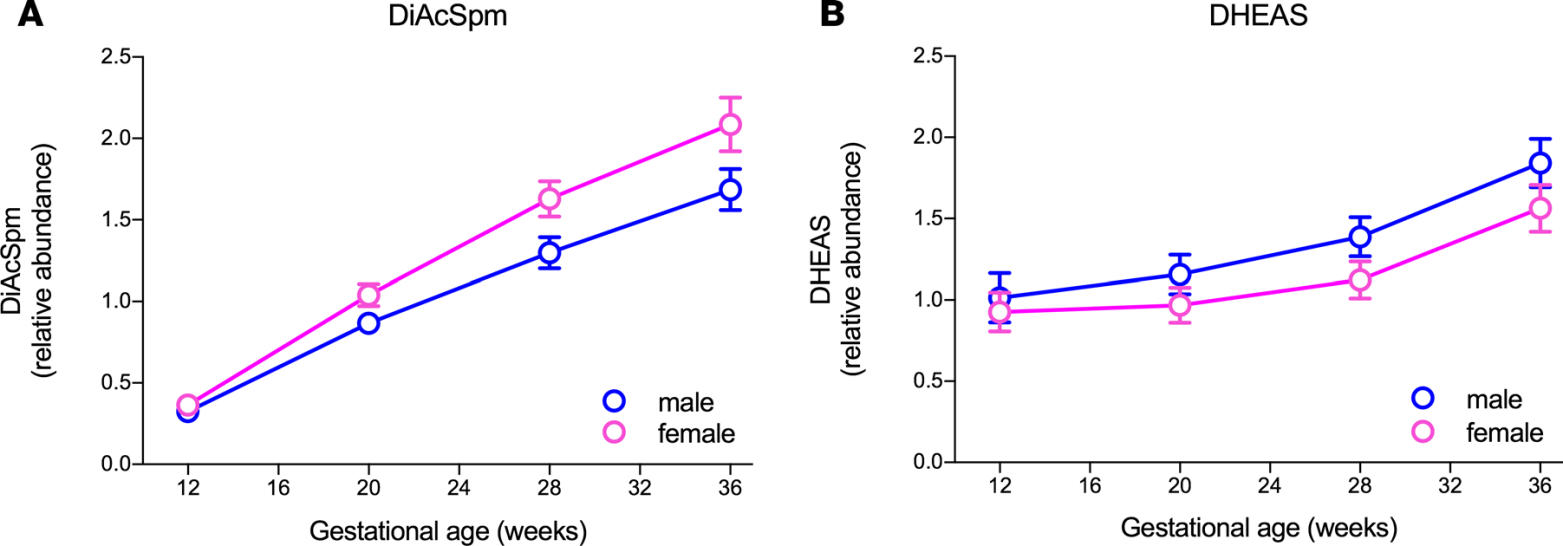
Maternal serum levels of DiAcSpm and DHEAS are regulated by fetal sex. Mean (points) with 95% CI (bars) at 12, 20, 28, and 36 wkGA for DiAcSpm (**A**) and DHEAS (**B**) comparing normal pregnancies with male and female fetuses. Pregnancies with female fetuses were *n* = 146 (12, 20, and 28 wkGA) and *n* = 131 (36 wkGA); pregnancies with male fetuses were *n* = 133 (12, 20, and 28 wkGA) and *n* = 128 (36 wkGA), respectively. Fetal sex differences at any of the first 3 gestational ages (composite hypothesis) were tested using a χ^2^ test and at 36 wkGA using a *t* test. Two-sided *P* value for the male/female difference was 0.0084 at 12, 20, and 28 wkGA and 0.0049 at 36 wkGA for DiAcSpm levels, and 0.0002 at 12, 20, and 28 wkGA and 0.0014 at 36 wkGA for DHEAS levels.

We next analyzed metabolomic data obtained using nontargeted ultra–high-performance liquid chromatography and tandem mass spectrometry (MS/MS) of serial serum samples collected at 12, 20, 28, and 36 weeks of gestational age (wkGA) from a random sample of healthy pregnant women from our cohort (*n* = 279) and compared the results in relation to fetal sex. The metabolomic profile quantified 837 metabolites of known structural identity. We performed mixed effects regression analysis of metabolite levels across the 12, 20, and 28 wkGA samples, a potential indicator of fetoplacental synthesis. We included fetal sex interactions with gestational age in the models to identify differences between the metabolite levels in relation to fetal sex (Table 2). We then validated candidate metabolites that appeared to vary by fetal sex using serum samples from the same women obtained at 36 wkGA. This approach identified 2 circulating metabolites with different maternal serum concentration according to the sex of the fetus: 16-OH-dehydroepiandrostenedione sulfate (DHEAS) was lower when the fetus was female, whereas N1,N12-diacetylspermine (DiAcSpm) was higher when the fetus was female (Figure 4). In both cases, the sex-related difference in maternal serum concentrations was consistent with increased mRNA levels of metabolic enzymes in the female placenta (Supplemental Table 3). Lower DHEAS in women pregnant with a girl was consistent with higher expression of steroid sulfatase (*STS*) in the female placenta. Higher DiAcSpm in women pregnant with a girl was consistent with higher placental expression of spermine synthase (*SMS*) in the female placenta. Both *STS* and *SMS* are X chromosome genes, and in the placenta, both escape from XCI (Supplemental Table 6).

**Figure 5:**
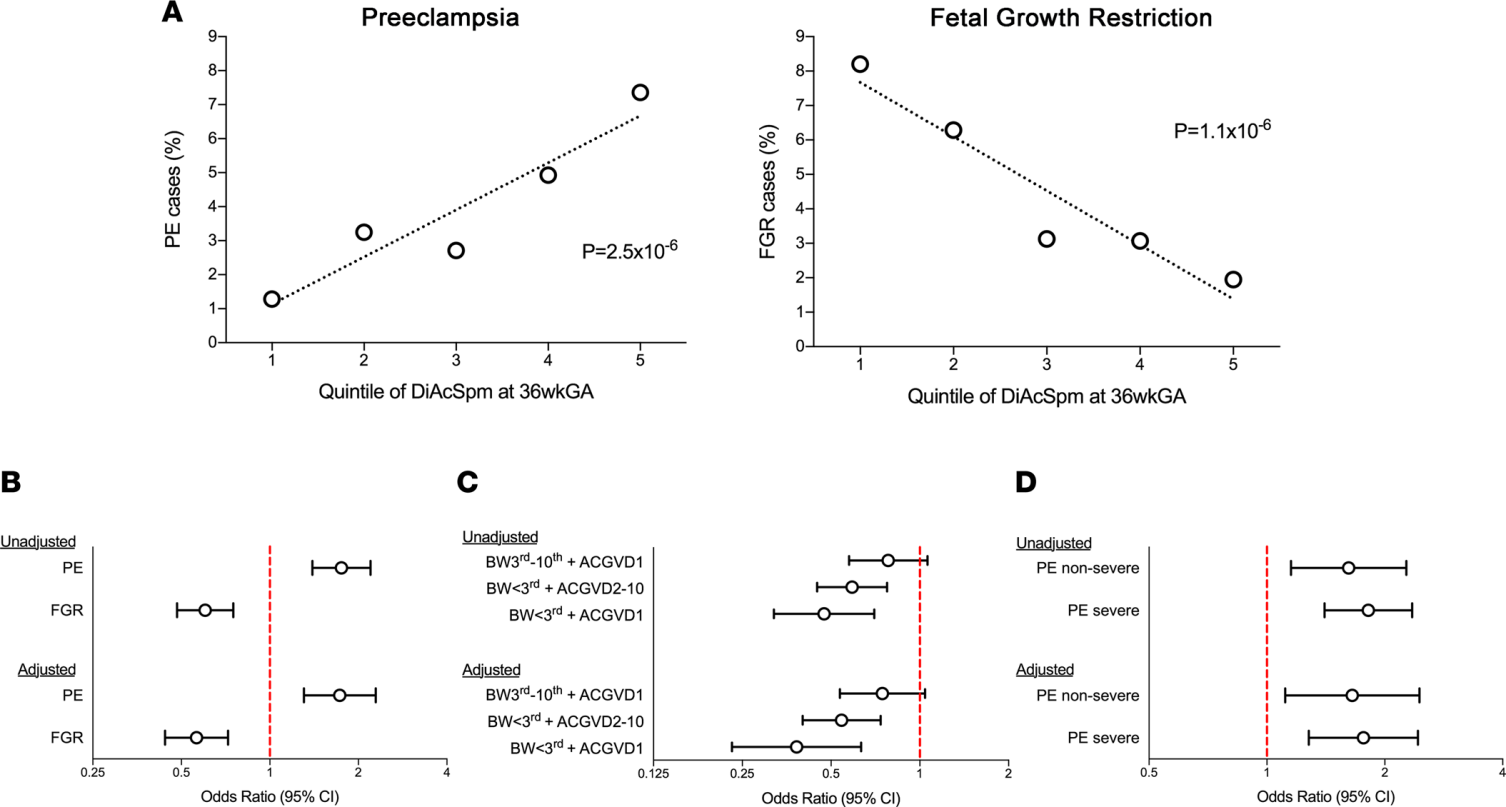
Maternal serum levels of DiAcSpm are regulated by pregnancy complications. (**A**) Proportion of cases with preeclampsia (including all severe cases and nonsevere nonsuperimposed cases as per ACOG 2013 definition; *n* = 134) or FGR (defined as customized birth weight [BW] centile [<3rd] or the combination of customized BW centile [<10th] and abdominal circumference growth velocity [ACGV] in the lowest decile [ACGVD1]; *n* = 162) by quintile of maternal serum DiAcSpm at 36 wkGA. *P* values for linear trend between the quintile and log-odds of each outcome (logistic regression) are reported. (**B**–**D**) Logistic regression modeling of the association between DiAcSpm (as a continuous variable, odds ratios [ORs] expressed for a 1 SD difference) at 36 wkGA and the risk of (**B**) preeclampsia (PE) and FGR, as defined in **A** (2-sided *P* < 0.001 for all ORs); (**C**) FGR stratified by phenotype, defined as the combination of (a) customized BW centile between the 3rd and the 10th and ACGVD1, (b) customized BW centile [<3rd] and ACGV deciles between the 2nd and 10th (ACGVD2-10), (c) customized BW centile [<3rd] and ACGVD1 (*n* = 52, *n* = 79, and *n* = 31, respectively; 2-sided unadjusted *P* = 0.11, *P* < 0.001, and *P* < 0.001, respectively; 2-sided adjusted *P* = 0.086, *P* < 0.001, and *P* < 0.001, respectively); (**D**) PE stratified to nonsevere and severe cases (*n* = 42 and *n* = 92, respectively; 2-sided unadjusted *P* = 0.005 and *P* < 0.001, respectively; 2-sided adjusted *P* = 0.013 and *P* = 0.001, respectively). Reported ORs are unadjusted and adjusted for sFLT1:PlGF at 36 wkGA, fetal sex, maternal age, height, BMI, ethnicity, and smoking status.

We then sought to determine whether the risk of placentally related complications of pregnancy varied according to maternal serum levels of the 2 sex-related metabolites. We compared the risk of subsequent delivery with a diagnosis of preeclampsia (*n* = 134) or subsequent delivery of a baby with FGR (*n* = 162), with reference to 259 controls, across quintiles of DHEAS and DiAcSpm. There was no association between maternal serum DHEAS and the risk of either outcome (Supplemental Figure 1). However, the risk of preeclampsia increased ~5-fold, and the risk of FGR declined ~5-fold across quintiles of maternal serum levels of DiAcSpm (Figure 5A). Both associations were highly statistically significant (*P* = 2.5 × 10^–6^ and 1.1 × 10^–6^, respectively) and were unaffected by adjustment for the soluble fms-like tyrosine kinase receptor 1 to placental growth factor ratio (sFLT1:PlGF, an existing placental biomarker for preeclampsia; refs. 21, 22), the sex of the fetus, and maternal characteristics (Figure 5, B–D). When the cases were stratified by phenotype, the association was strongest in the group of most severe FGR (Figure 5C). In contrast, the association did not differ comparing severe and nonsevere preeclampsia (Figure 5D). Collectively, the multiomics approach identified the polyamine pathway as differing by fetal sex and being differentially associated with the 2 major placentally related complications of human pregnancy. Therefore, we next set out to study this pathway to determine whether there was functional evidence indicating a role in sex-related differences in the human placenta.

### Sex-related differences in the effects of polyamine depletion in trophoblast cells.

**Figure 6:**
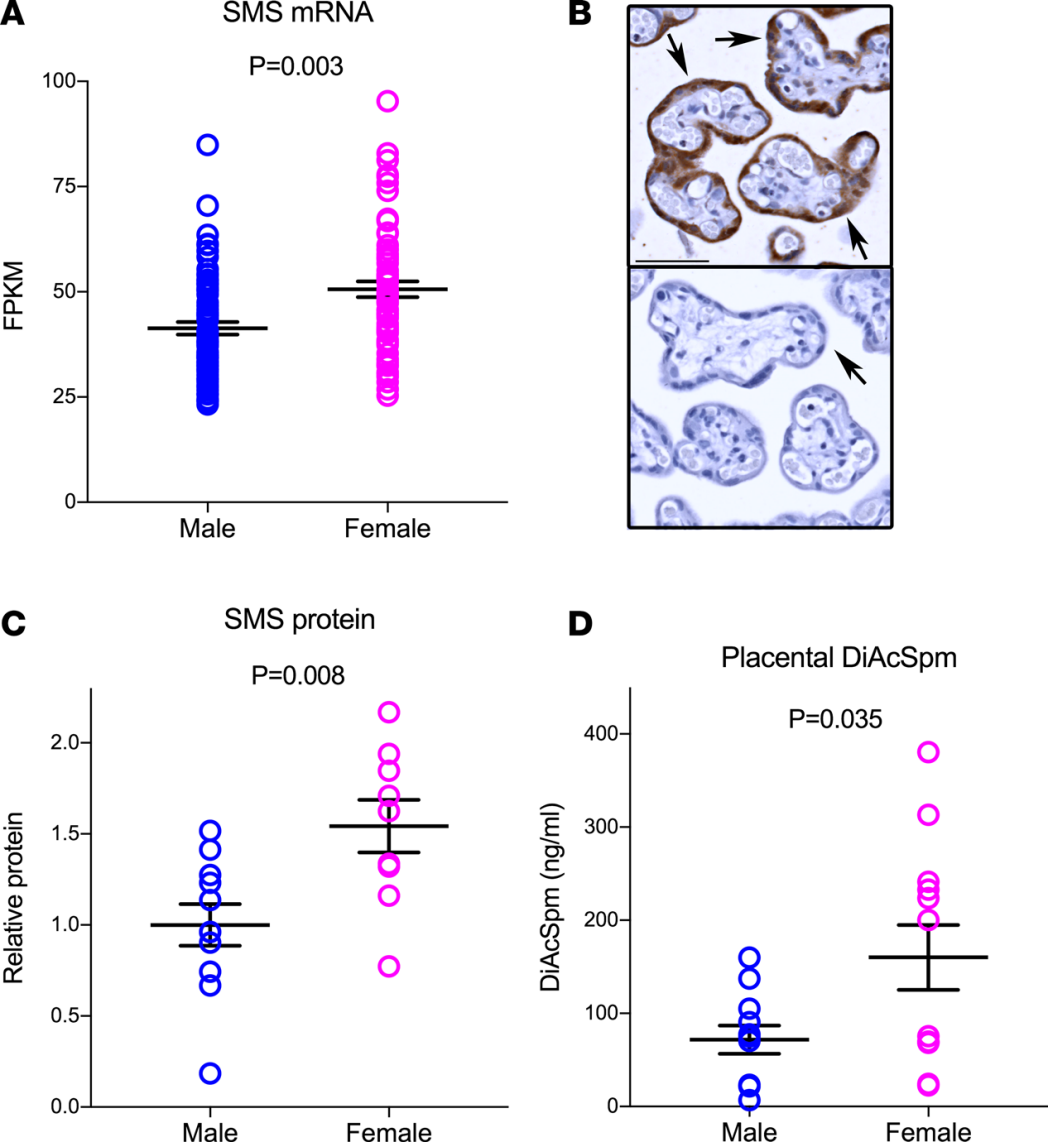
Higher spermine synthase expression in female placentas. (**A**) SMS transcript level in male (*n* = 67) and female (*n* = 64) placental tissues from healthy individuals. FPKM, fragments per kilobase of transcript per million mapped reads. (**B**) Immunohistochemical localization of SMS in term human placenta (top panel: rabbit anti-SMS; lower panel: rabbit IgG-control). Tissues obtained from 3 different placentas. Arrows indicate the placental trophoblast cell layer. Scale bar: 50 μm. (**C**) Western blot analysis of SMS protein in male (*n* = 11) and female (*n* = 9) primary trophoblast cells. Mean ± SEM. Student’s unpaired *t* test. (**D**) LC-MS analysis of DiAcSpm in male and female placental tissues (*n* = 12 in each group). Mean ± SEM. Student’s unpaired *t* test.

**Figure 7:**
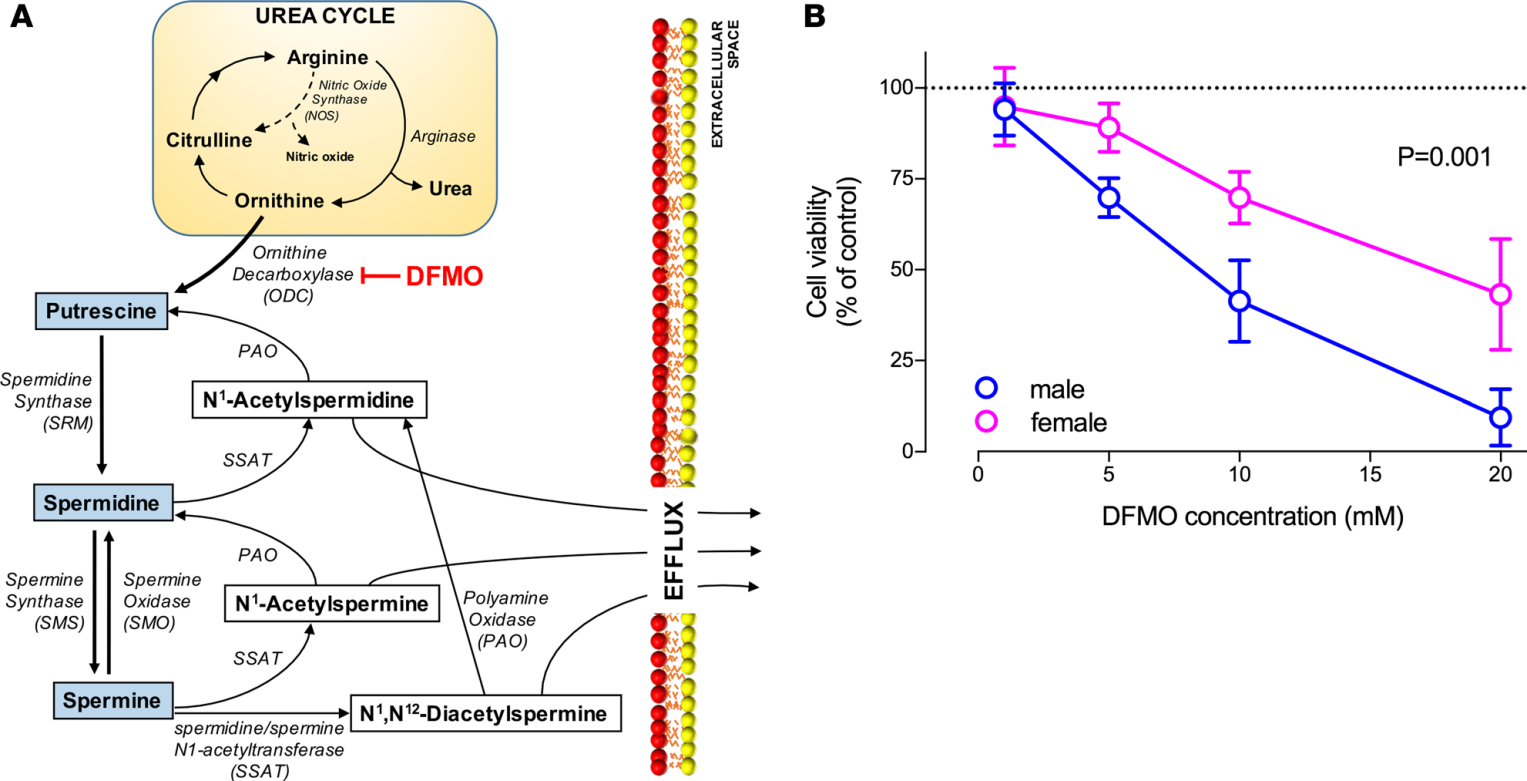
Polyamine depletion effects on primary trophoblast cells viability. (**A**) Schematic representation of the polyamine metabolic pathway. (**B**) Effect of the ornithine decarboxylase inhibitor, difluoromethylornithine (DFMO), on the viability of trophoblast cells isolated from male (*n* = 9) and female (*n* = 9) placentas. Points, mean; bars, 95% CI. The interaction between fetal sex and the change in DFMO concentration on cell viability was estimated using a longitudinal linear mixed model with the Kenward-Roger denominator degrees of freedom method; a 2-sided exact *P* value accounting for small-sample bias was obtained.

The RNA-seq data indicated that *SMS* mRNA was ~25% higher in the female placenta compared with the male (Figure 6A and Supplemental Table 3). IHC localized the protein to the trophoblast layer of the placental villi (Figure 6B), and we confirmed higher levels of SMS protein in females using Western blot analysis of primary trophoblast cells (Figure 6C). We performed liquid chromatography–mass spectrometry (LC-MS) and studied the concentration of polyamines and their acetylated metabolites in relation to fetoplacental sex. The concentration of DiAcSpm was higher in the female placenta, consistent with the maternal serum metabolomics (Figure 6D). The functional importance of sex-related differences in placental polyamine metabolism was assessed by comparing the effect of the global polyamine synthesis inhibitor difluoromethylornithine (DFMO) on the viability of primary cultures of male versus female trophoblast cells. We found that male trophoblast cells were more sensitive to this drug than female cells (Figure 7, A and B). This demonstrated that the placenta-specific escape of *SMS* from XCI was associated with lower levels of SMS protein and SMS metabolites in male trophoblast cells and that male cells were more susceptible to the harmful effects of polyamine depletion.

## Discussion

The key findings we report here are that differences in placental polyamine synthesis exist in male and female placentas and that this pathway is implicated in the pathophysiology of placentally related complications of human pregnancy. We used a multiomic approach to identify this pathway. We studied the placenta using RNA-seq and oxidative bisulfite DNA-seq, and we studied maternal serum using metabolomics. This approach identified 2 pathways that differed according to fetal sex. We then compared the levels of the associated metabolites in maternal serum in relation to the risk of preeclampsia and fetal growth restriction. A single metabolite, DiAcSpm, was found to differ both in relation to fetal sex and the risk of placentally related complications of pregnancy. We then performed targeted experiments using primary culture of human trophoblast cells, which confirmed sex-related differences in the cellular effects of pharmacological interruption of the pathway. We conclude that placental sex is associated with differences in polyamine metabolism and that this variation may contribute to the known associations between fetoplacental sex and the “great obstetrical syndromes” (1).

Preeclampsia and FGR share a number of associations with biomarkers of placental function, including low first trimester of pregnancy-associated plasma protein A (PAPP-A), high second trimester α-fetoprotein (AFP) in maternal serum (23), and high resistance patterns of uterine artery Doppler flow velocimetry (24, 25). However, DiAcSpm is the first circulating maternal factor to our knowledge to demonstrate opposite associations with the 2 conditions. In the present analysis, 2 serum metabolites were identified from the 837 known metabolites included in the assay. The basis for their selection was very strong statistical evidence that they varied by sex. The probability that, in the absence of association between DiAcSpm and the 2 complications (preeclampsia and FGR), we would have produced results suggesting an association this strong or stronger was in the region of 1 in 1,000,000 for both outcomes, far below the conventional 1 in 20 threshold for statistical significance. Moreover, the 5-fold variation in the risk of each outcome comparing women with the lowest 20% of serum levels with women with the highest 20% of serum levels reflects a clinically important and biologically meaningful difference in risk. Our previous studies of the cohort have demonstrated that the maternal serum sFLT1:PlGF ratio is elevated at 36 wkGA in women who subsequently experience either condition, i.e., preeclampsia or FGR without any hypertensive complications (21, 26). The ratio is thought to reflect placental oxidative stress, which is considered to be a feature of both conditions. The fact that the association between maternal serum levels of DiAcSpm was positive for preeclampsia but negative for FGR suggests that the ratio does not merely reflect some underlying feature common to both conditions, such as placental hypoxia. Indeed, the opposite associations suggest that the polyamine pathway may determine whether placental dysfunction results in preeclampsia, FGR, or both conditions concurrently. Unfortunately, the number of cases affected by both FGR and preeclampsia where metabolomics data were also available was too small for meaningful analysis. Similarly, there were insufficient numbers of intrauterine fetal deaths for study, and both of these are areas for future research. It also remains to be established whether any effects are local to the placenta or reflect systemic effects on the mother. A protective effect of SMS activity on FGR is supported by the phenotype of SMS deficiency in humans (Snyder-Robinson syndrome), where the average birth weight (BW) of babies born at term (27, 28) was ~500 g less than the population average of 3,300 g (29).

The mechanisms leading to XCI are still only partially understood. The causes of tissue specificity also remain obscure. The fact that a gene might escape XCI in one tissue but not in another suggests that there might be tissue-specific regulatory factors. The majority of the genes that escaped XCI in the placenta are associated with transcriptional and translational modifications. These include zinc finger proteins (*ZFX*, *ZRSR2*, *ZMAT1*) and histone deacetylases (*KDM5C*, *KDM6A*, *HDAC8*), which regulate gene expression, as well as eukaryotic initiation factors (*EIF2S3*, *EIFAX*) and ribosomal proteins (*RPS4X*), which mediate protein translation. This suggests that genes that escape XCI may drive placental sex–specific responses under basal conditions or in response to environmental stimuli. However, further studies will have to address why, for example, *SMS* escapes XCI in the placenta but not in other tissues. Interestingly, we have recently shown sex-related differences in autosomal genes, where differential methylation of CUB And Sushi Multiple Domains 1 (*CSMD1*) was associated with a placenta-specific transcript and a placenta-specific sex-related difference in mRNA levels (30). Better understanding of the determinants of sex-related differences in the placenta might shed further light on key pathways involved in the pathophysiology of disease.

The current findings can also be interpreted in the light of current theories in evolutionary biology. Trivers and Willard postulated that there is a survival advantage to investment of resources in female over male offspring when resources are constrained in pregnancy (31). Our findings could be interpreted in light of this hypothesis — specifically, that placental escape of the *SMS* gene from XCI may enhance polyamine metabolism and confer a survival advantage to female fetuses developing with suboptimal placental function, given our discovery of a protective effect of higher DiAcSpm levels on FGR. The finding of associations in opposite directions between DiAcSpm and preeclampsia (positive association) and DiAcSpm and FGR (negative association) is particularly interesting. The 2 conditions share many common features, but it is currently unclear why placental dysfunction can lead to preeclampsia without FGR or FGR without preeclampsia. The current data indicate that placental polyamine metabolism may have a role. Haig (32) hypothesized that preeclampsia may be an adaptive response that allows the fetus to increase its share of resources by inducing maternal hypertension. This hypothesis would predict the existence of factors that increase the risk of preeclampsia but decrease the risk of FGR, and maternal serum DiAcSpm demonstrated exactly that pattern of association.

Polyamines (putrescine, spermidine, and spermine) are involved in multiple key processes implicated in many disease states, including stem cell function, autophagy, tumor suppression, immunoregulation, and both cardio- and neuro-protection (reviewed in ref. 33). Polyamines are synthesized by cells from amino acid precursors, but other sources include dietary intake and the intestinal microbiota. Dietary supplementation of mice with spermidine increased longevity (34), and the potential benefits of dietary supplementation with polyamines is an area of intense interest in multiple pathological conditions. Interestingly, the human placenta had the highest ratio of SMS to spermidine synthase activity when compared across a range of human tissues (35). However, we provide the first evidence to our knowledge that the placenta exhibits a unique sex-dependent difference in polyamine metabolism, which is associated with placental-specific escape from XCI by *SMS*. Given our findings that maternal serum levels of polyamine metabolites differ by fetal sex and strongly differentially associate with the great obstetrical syndromes, understanding the effects of polyamine metabolism on placental function is an important area for future research. These associations could be of practical significance through the potential for dietary manipulation of polyamine metabolism.

## Methods

### Study design

The Pregnancy Outcome Prediction (POP) study was conducted at the Rosie Hospital (Cambridge, United Kingdom), as previously described (13, 36). In brief, it was a prospective cohort study of nulliparous women attending the hospital for their dating ultrasound scan between January 14, 2008, and July 31, 2012, with a viable singleton pregnancy. Women had blood taken at the booking visit (~12 wkGA) and at 3 subsequent visits at the NIHR Cambridge Clinical Research Facility (at ~20 wkGA, ~28 wkGA, and ~36 wkGA), when an ultrasound scan was also performed. Patients and clinicians were blinded to the results of the research ultrasound scans. The characteristics of the study cohort (*n* = 4,212) by outcome status are described in Table 1.

### RNA-seq data processing and analysis

Extraction of total RNA from placental biopsies and library preparation for RNA-seq are described in the Supplemental Methods.

#### Alignment of reads and quantification of transcript abundance.

Reads were trimmed using cutadapt (37) and Trim Galore! (38) and were mapped to the GRCh37 (hg19) version of human genome reference. The so-called 2-pass (or 2-scan) alignment protocol was applied to rescue unmapped reads from the initial mapping step using TopHat2 (39), a splice-aware mapper built on top of Bowtie2 short-read aligner (40, 41). The initial and second mapped reads were merged by SAMtools (42), and the uniquely mapped reads were counted by the htseq-count of HTSeq (43) using the ENSEMBL version 75 (GENCODE 19) transcriptome definition. Transcript abundance was measured in fragments per kilobase of transcript per million mapped reads (FPKM), and the expression fold change was measured using DESeq2 Bioconductor package (44).

#### Identification of sex-biased genes and tissue-wide comparison.

The RNA-seq data from GTEx analysis version V6p (16) were downloaded from https://gtexportal.org/home/datasets Samples satisfying the following attributes were used: (a) mapping rate (SMMAPRT) > 0.9; (b) exonic rate (SMEXNCRT) > 0.8; and (c) mean coverage per-base (SMMNCPB) > 20. Tissues having at least 20 qualifying samples of both sexes were used, and tissue subtypes were manually selected if 2 or more were available. Additionally, sex-specific tissues were removed from the analysis: cervix, fallopian tube, ovary, prostate, testis, uterus, vagina, and breast. A total of 3,077 GTEx sample IDs selected in the final 19 tissues were analyzed in this study. Our and the GTEx data were analyzed applying the same settings in DESeq2. Chromosome Y genes were excluded from the analysis. We identified female-biased genes on chromosome X meeting the following criteria: (a) mean read-count > 10, (b) fold change > 10% (i.e., more than 10% increase in females compared with males), and (c) Benjamini-Hochberg (45) adjusted *P* < 0.01 (corrected for the number of the chromosome X genes from the original *P* values). The X chromosome genes with sex-biased expression in placental and the 19 GTEx tissues included in this study are listed in Supplemental Table 3.

### Placental methylome analysis

DNA extraction from placental biopsies is described in the Supplemental Methods.

#### Whole genome oxidative bisulfite sequencing (WGoxBS).

Placental genomic DNA samples (4 μg) from 4 healthy pregnancies (*n* = 2/each fetal sex) were fragmented into 10 Kb fragments with the g-TUBE (Covaris) and concentrated using GeneJET purification columns (Thermo Fisher Scientific). Part of this fragmented DNA (1.5 μg/sample) was taken forward for oxidation + bisulfite treatment using the TrueMethyl kit (Cambridge Epigenetix). Bisulfite converted DNA (50 ng/sample) was used as input for library generation using the EpiGnome Methyl-Seq Kit (Epicentre) and EpiGnome Index PCR Primers (Epicentre). Indexed libraries were pooled in an equimolar ratio. To increase the number of uniquely sequenced reads, 2 independent libraries were generated for each individual. Multiplexed sequencing was carried out on the Illumina MiSeq and HiSeq2500 instruments with 2 × 100, 2 × 150 and 2 × 125 cycles using MiSeq Reagent Kit v3, HiSeq SBS kit v3, and HiSeq SBS kit v4, respectively. The methylation conversion rate was checked with bsExpress (https://bitbucket.org/cegx-bfx/cegx_bsexpress_docker).

#### In-solution target capture library preparation and sequencing.

Placental genomic DNA (3.5 μg) was extracted from 8 healthy pregnancies. The samples were *n* = 4/each fetal sex and included 2 samples analyzed by WGoxBS. Therefore, 2 samples represented a technical validation experiment, and 6 samples represented a biological/technical validation experiment (using a different technique and another set of samples). The genomic DNA was fragmented using the Covaris S220 system and processed according to the SureSelect Methyl-Seq target enrichment protocol (Agilent). All 8 libraries were pooled and sequenced on 1 lane of the Illumina HiSeq2500 (2 × 125 cycles using HiSeq SBS kit v4) and 1 lane of the Illumina HiSeq4000 (2 × 150 cycles using HiSeq 3000/4000 SBS kit) following Illumina’s guidelines.

#### Analysis of methylation sequencing data.

WGoxBS and in-solution target capture data had adapter sequences removed and the quality assessed using Trim Galore! and FastQC (46), respectively. High-quality trimmed reads (i.e., base quality ≥ 20) were mapped to the human genome reference (hg19) using Bismark (47). After removing reads with poor mapping quality (MAPQ = 0) and duplicated reads, methylated CpGs were called using Bis-SNP (48). CpGs with at least 10× coverage were considered in the analysis. The summary statistics for the WGoxBS and in-solution target capture data processing are available in Supplemental Table 5. The methylation levels of individual CpG sites were measured by the ratio of reads with methylation (i.e., C-base reads) out of the total number of reads covering the position (i.e., sum of C and T reads). The methylation levels of promoter regions (1,000 bp upstream and 1,000 bp downstream regions of the transcript start sites) were measured if there were at least 4 CpGs (≥10×) by weighting individual methylation levels as follows: the sum of reads with methylation out of the total number of reads over the promoter region. Sex-specific methylation differences at the promoter regions of chromosome X genes are available in Supplemental Table 6. Promoter methylation analysis of X chromosome genes in male and female placentas was performed comparing genes subject to XCI, escaped from XCI (divided in genes uniquely identified in placenta or identified in at least 1 of 19 GTEx tissues), and with male-biased expression. Statistical analysis was performed using Mann-Whitney *U* tests and Kruskal-Wallis rank tests.

### Primary human trophoblast cell culture, Western blotting of SMS, and DFMO treatments

Human placental tissues were collected from healthy women with normal-term pregnancies and scheduled for delivery by elective cesarean section following written informed consent. Primary trophoblast cells were isolated as described in the Supplemental Methods.

Cells were harvested for Western blot analysis of SMS at 90 hours, after differentiation into syncytiotrophoblasts (Supplemental Methods). Membranes were probed with rabbit anti-SMS primary antibody (0.5 μg/ml, Abcam, ab156879) and peroxidase-conjugated goat anti-rabbit secondary antibody (1:2000, Cell Signaling Technology, 7074), followed by β-actin (0.1 μg/ml, Abcam, ab8229). Target protein level was normalized to β-actin (Image J software), and the mean density of bands from male samples were assigned an arbitrary value of 1. Membranes were also stained for total protein using Amido Black Stain (Sigma). The results did not differ when corrected for β-actin or total protein staining (data not shown).

To analyze the effects of polyamine depletion on cell viability, after 66 hours in culture, trophoblast cells were incubated for 24 hours in the presence of DFMO (1–20mM) or vehicle (H_2_O) in media with reduced serum (1% FBS) and 1 mM aminoguanidine to inhibit serum amine oxidase activity (49). The viability of trophoblast cells was determined by their ability to metabolize 4,5-dimethylthiazol-2-yl-2,5-diphenyltetrazolium bromide (MTT, MilliporeSigma). Briefly, cells were incubated with 1 mg/ml of MTT reconstituted in PBS for 4 hours at 37°C, lysed with 10% SDS, and absorbance read at 570 nm.

### SMS IHC in human term placenta

IHC was performed on formalin-fixed, paraffin-embedded placental sections as described in the Supplemental Methods, using rabbit anti-SMS (1:20 dilution) or anti–rabbit IgG (1:20 dilution, Abcam, ab172730), followed by biotin-conjugated anti rabbit secondary antibodies (1:200, Dako, E0432) and Vectastain ABC kit (Vector Labs, PK-6100).

### LC-MS

LC-MS was performed on placental biopsies lysed in PBS and 10% of spermidine-(butyl-d8) trihydrochloride internal standard (Supplemental Methods). Chromatographic separation was achieved using an ACE Excel 2 C18-PFP LC-column with a Shimadzu UPLC system (Shimadzu UK Limited). MS detection was performed on an Exactive orbitrap mass spectrometer (Thermo Fisher Scientific) operating in positive ion mode.

### Serum metabolomic profiling

#### Measurements and analyses.

Serum metabolites were measured at Metabolon Inc. by nontargeted ultra–high-performance LC-MS and MS/MS (50), resulting in the identification of 837 compounds of known structural identity. Peaks were quantified using AUC of primary MS ions. Samples were run in batches of 36, and all batches contained the same proportions of cases and controls. All samples from a given woman were run in the same batch. Missing values were assumed to be the result of falling below the detection sensitivity and, thus, were imputed with the minimum detection value based on each metabolite. To adjust for instrument batch effects for each run day, the raw ion counts for each metabolite were divided by the median value for the run day. Hence, metabolites were expressed as multiples of the median, and we called this relative abundance in Figure 4.

A case-cohort design was applied (see below). Sex differences in the metabolomic profiles were assessed in a random subcohort, excluding cases (*n* = 279). We fitted longitudinal linear mixed models to identify metabolites that changed in the maternal circulation with advancing gestational age up to 28 wkGA differentially by fetal sex. Scaled imputed metabolite values (multiples of the median) were log-transformed before the analysis. We chose the top 10 metabolites differing by fetal sex with the lowest *P* values from the composite χ^2^ test (2-sided). We then validated the result using the metabolite level at 36 wkGA from the same women by fitting a regression model between the metabolite and fetal sex. We selected the metabolites with validation *P* < 0.01 for further analysis (Table 2). The distributions of the selected metabolites (DiAcSpm and DHEAS) were examined in noncases, and the best transformations were applied. Quintiles of DiAcSpm and DHEAS were calculated for all women at 36 wkGA, based on the distribution in the noncases. The association between the outcomes and the quintile of each metabolite was analyzed using logistic regression. The quintile was included in the model (a) as a categorical and (b) as a continuous exposure. All associations between the metabolite and log-odds of the outcome were approximately linear, and a 2-sided *P* value for linear trend was reported. To obtain the proportion of cases, the noncases were weighted by the inverse of the random subcohort sampling fraction (4,177/325 = 12.85). Next, both metabolites were analyzed as continuous variables. The transformed variables were turned into Z scores. Logistic regression models were fitted between both metabolites at 36 wkGA and the outcome, with and without adjustments for maternal and fetal characteristics and the sFLT1:PlGF ratio (Supplemental Methods). Similar analyses were performed on maternal serum levels of spermidine (see Supplemental Figure 2, showing no sex or disease associations with spermidine levels), while spermine was not included in the metabolomic panel.

#### Description of the case-cohort design used in the metabolite analysis.

In total, 4,212 women completed the POP study. After excluding miscarriages, fetal deaths prior to 23 weeks and terminations (*n* = 29), and women who did not have any blood samples for analysis (*n* = 6), 4,177 women remained in the cohort.

Ten groups of cases were defined, but only the preeclampsia and FGR cases born at term were included in the present study. The groups were (a) preeclampsia with preterm delivery; (b) customized BW centile (<10th) (51) with preterm delivery; (c) gestational diabetes requiring drug treatment; (d) gestational diabetes requiring diet treatment; (e) spontaneous preterm delivery; (f) severe, nonsuperimposed preeclampsia, term delivery; (g) customized BW centile (<10th) with abdominal circumference growth velocity (ACGV) in the lowest decile between 20 and 36 wkGA (ACGVD1) (52); (h) severe, superimposed preeclampsia, term delivery; (i) nonsevere, nonsuperimposed preeclampsia, term delivery; and (j) customized BW centile (<3rd) delivered at term. In total, 644 cases belonged to 1 or more of these groups. For the present study, preeclampsia was defined by combining groups f, h, and i (any severe preeclampsia or nonsevere nonsuperimposed preeclampsia), as per the ACOG 2013 definition (21), and FGR was defined by combining groups g and j (customized BW centile [<3rd] or the combination of customized BW centile [<10th] and ACGVD1). Preterm births were excluded. A subcohort of 325 women was randomly selected in the cohort, and 46 of these women developed 1 or more of the 10 outcomes. The remaining 279 healthy women were used in the analysis assessing the changes in metabolites levels between 12 wkGA and 28 wkGA. The analysis of preeclampsia and FGR was performed among women who had the metabolite measurement available at 36 wkGA and gave birth at term. The analysis of the outcomes included 134 cases of preeclampsia, 162 cases of FGR (9 of these had also preeclampsia), and a shared comparison group of 259 healthy women. In a further analysis, we stratified FGR by phenotype, defined as the combination of (a) customized BW centile between the 3rd and the 10th and ACGVD1, (b) customized BW centile (<3rd) and ACGV deciles between the 2nd and 10th (ACGVD2-10), and (c) customized BW centile [<3rd] and ACGVD1 (*n* = 52, *n* = 79 and *n* = 31, respectively). Preeclampsia was stratified to nonsevere and severe cases (*n* = 42 and *n* = 92, respectively).

### Software and data availability

The software used in this study is listed in Supplemental Table 7. All the computational analyses were conducted using the Linux clusters at the University of Cambridge High Performance Computing Service and the Linux workstations of School of Biological Sciences computing service. The data sets generated during the current study have been deposited in the EGA repository (https://www.ebi.ac.uk/ega/) under ID codes DNA methylation data, EGAD00001003136; RNA-seq data, EGAD00001003457.

### Statistics

Statistical analyses were performed using Stata 14.2 (StataCorp). All *P* values were 2-tailed, and statistical significance was assumed at *P* < 0.05, unless otherwise specified. Specific statistical methods are further described in details in the relevant sections of Methods.

### Study approval

Ethical approval was given by the Cambridgeshire 2 Research Ethics Committee (reference number 07/H0308/163), and all participants provided written informed consent.

## Author contributions

RNA-seq was contributed by FG and JD. Bioinformatics were contributed by SG, JD, and MDJ. Methylation experiments were contributed by MDJ. Statistics were contributed by US and AMW. Cell culture and immunohistochemistry were contributed by ILMHA. Technical assistance was contributed by EC. LC-MS experiments were contributed by BJJ and AK. Supervision was contributed by RAC, MC, DSCJ, and GCSS. Writing was contributed by GCSS. Manuscript approval was contributed by all authors.

## Supplementary Material

Supplemental data

Supplemental Tables 1-7

## Figures and Tables

**Table 2:**
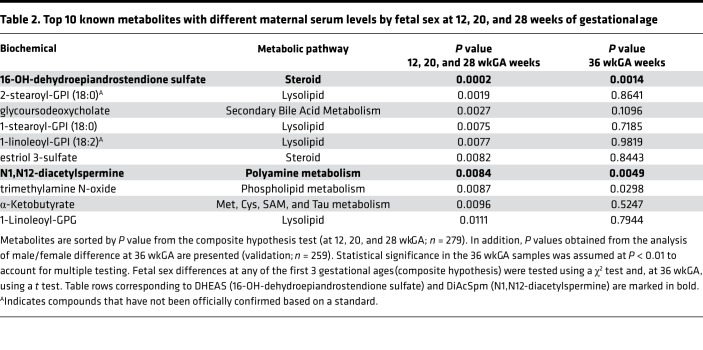
Top 10 known metabolites with different maternal serum levels by fetal sex at 12, 20, and 28 weeks of gestational age

**Table 1:**
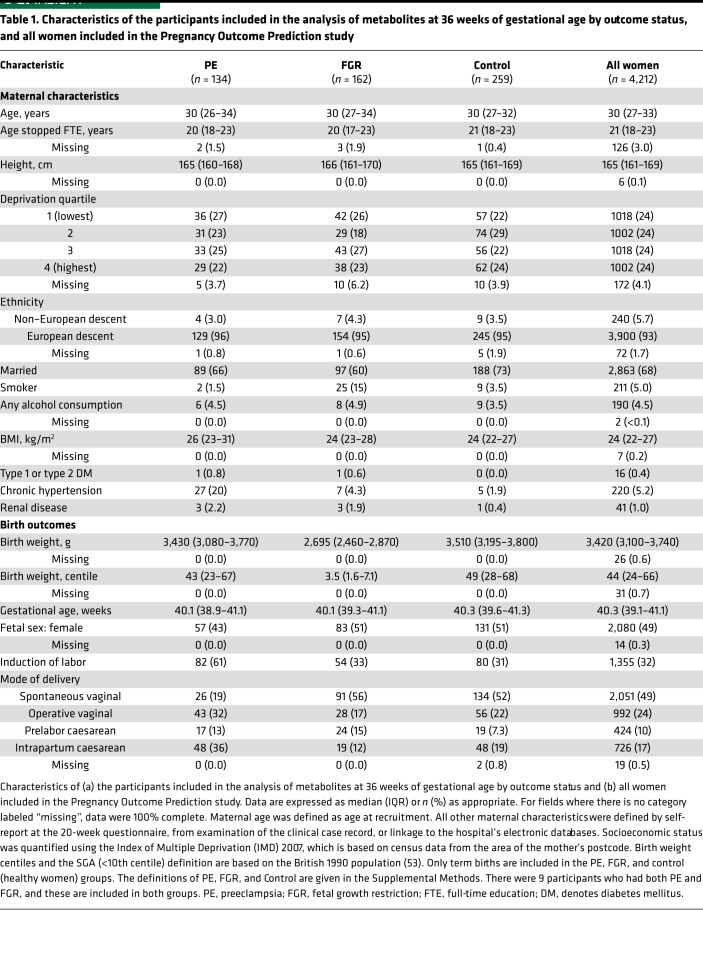
Characteristics of the participants included in the analysis of metabolites at 36 weeks of gestational age by outcome status, and all women included in the Pregnancy Outcome Prediction study

## Version 1 07/12/2018

Electronic publication
