# Supplementary material for: Placental polyamine metabolism differs by fetal sex, fetal growth restriction, and preeclampsia
Source: JCI Insight. 2018 Jul 12;3(13):e120723. doi: 10.1172/jci.insight.120723 (PMC6124516; doi:10.1172/jci.insight.120723)
Supplement: Supplemental data [file jciinsight-3-120723-s221.pdf]

Supplemental Material

Supplemental Methods

POP study outcome data

Outcome data were ascertained by review of each woman's paper case record by research midwives and by record linkage to clinical electronic databases of ultrasonography (Astraia, Munchen, Germany), delivery (Protos, iSoft, Banbury, UK), biochemical tests (Meditech, Westwood MA, USA) and neonatal intensive care (Badgernet, Clevermed Ltd, Edinburgh, UK). Where preeclampsia was suspected, there was a second review of the clinical case record to confirm the diagnosis and classification on the basis of the objective criteria of the 2013 ACOG Guideline (1).

Maternal serum immunoassays

Maternal circulating levels of sFLT1 and PlGF were measured in all serum samples collected in the POP study using Roche Elecsys assays on the electro-chemiluminescence immunoassay platform, Cobas e411 (Roche Diagnostics) (2). With this system, the intra-assay coefficient of variation for human serum samples is <2% for both the assays, and the inter-assay coefficients of variation are 2.3-4.3% for the sFLT1 assay and 2.7-4.1% for the PlGF assay. Researchers performing the assays were blinded to the patients' clinical information and pregnancy outcome.

Placental biopsies, RNA extraction and library preparation for RNA-seq

Placental biopsies were obtained as part of the POP study from 134 healthy pregnancies. Subsequently, 3 samples were excluded due to the presence of decidual contamination of the placental biopsies. These samples had 20-fold higher transcript levels of the following decidual genes compared to the median value: insulin like growth factor binding protein 1 (IGFBP1), osteomodulin (OMD), prolactin (PRL), retinol binding protein 4 (RBP4), KIAA1644, RAR related

orphan receptor B (RORB) and chordin like 1 (CHRDL1) (3, 4). Therefore, the final analysis was performed on 131 samples from healthy pregnancies with male (n=67) and female (n=64) fetuses. Control patients delivered live babies with a birth weight percentile in the normal range (customized and non-customized birth weight 20-80th percentile (5)) with no evidence of slowing in fetal growth trajectories and had no evidence of hypertension at booking and during pregnancy, did not experience preeclampsia, Hemolysis/Elevated Liver enzymes/Low Platelet (HELLP) syndrome, gestational diabetes or diabetes mellitus type I or type II or other obstetric complications. For each biopsy total placental RNA was extracted from approximately 5 mg of tissue using the "mirVana miRNA Isolation Kit" (Ambion) followed by DNase treatment ("DNA-free DNA Removal Kit", Ambion). Libraries were prepared from 300-500ng of total placental RNA (RIN values ≥ 7.0) with the "TruSeq Stranded Total RNA Library Prep Kit with Ribo-Zero Human/Mouse/Rat" (Illumina), pooled and sequenced (single-end, 125bp) using a Single End V4 cluster kit and Illumina HiSeq2500 and HiSeq4000 instruments. Sequencing was to a depth of ~100 million unmapped reads per sample (Supplemental Table 1).

DNA extraction for placental methylome analysis

A portion from each of the four biopsies previously collected from each placenta were combined to provide a single 20-24mg sample. DNA was extracted using the Qiagen DNeasy Blood & Tissue Kit (Qiagen) with RNase treatment and quantified using the Qubit dsDNA HS Assay Kit (Life Technologies).

Primary human trophoblast cell isolation and harvesting for western blot analysis

Trophoblast cells were isolated from human placental tissues by trypsin digestion and Percoll purification as previously described (6). Briefly, approximately 40g of villous tissue digested in trypsin (0.25%, Gibco) and DNAse I (325 Kunits/mg tissue, Sigma) and purified over a discontinuous 10–70% Percoll gradient centrifugation. Cells which migrated between 35–55% Percoll layers were collected and cultured in 1:1 mixture of Dulbecco's modified Eagle's medium

(Sigma-Aldrich) and Ham's F-12 nutrient mixture (Gibco) containing 10% fetal bovine serum, 50µg/ml gentamicin, 60µg/ml benzyl penicillin and 100µg/ml streptomycin (Sigma), and incubated in a 5% CO_2 humidified atmosphere at 37°C. Following 18 hours of culture, attached cells were washed twice in warmed Dulbecco's PBS and culture media was changed daily.

After 90 hours in culture, cells were harvested for Western blot analysis using RIPA buffer (50mM Tris HCl, pH=7.4; 150mM NaCl; 0.1% SDS; 0.5% Na-deoxycholate and 1% Triton X100) containing protease inhibitors and phosphatase inhibitor cocktail 1 and 2 (1:100, Sigma).

Spermine synthase immunohistochemistry in human term placenta

Formalin-fixed, paraffin-embedded placental sections (7µm) were deparaffinized before undergoing antigen retrieval in sodium citrate buffer. After the quenching (5% H_2O_2) and blocking (5% normal goat serum) steps, sections were incubated with primary and secondary antibodies and Vectastain ABC kit (Vectorlabs) as described in the Methods. Peroxidase detection was performed using SIGMAFAST DAB tablets (Sigma, #D4293). Sections were then counterstained with 50% Gill No.3 hematoxylin. Digital images of immunostained slides were obtained using the Nanozoomer slide scanner (Hamatsu Photonics) at 40X magnification. Immunohistochemistry was performed using sections from three different placentas.

Liquid chromatography–mass spectrometry (LC-MS)

Placental biopsies (~10mg) were suspended in 180µL of PBS and 20µL of spermidine-(butyl-d8) trihydrochloride internal standard. After adding 500µL of cold acetonitrile, samples were homogenized using a Bioprep-24-1004 homogenizer (Allsheng). The samples were then centrifuged at 21,000g for 5 minutes and the supernatants were separated and dried down under oxygen free nitrogen. To reconstitute the polyamines, 200µL of 0.1% formic acid was added to the dried extract. Chromatographic separation and mass spectrometry detection are described in the Methods.

Supplemental Tables

Supplemental tables provided in excel file:

Supplemental Table 1. Mapping statistics for RNA-seq experiments (male and female placental tissues).

Supplemental Table 2. Differentially expressed genes (DEGs) in male and female placentas.

Supplemental Table 3. X-chromosome genes with sex-biased expression in the placenta and the 19 GTEx tissues.

Supplemental Table 4. Expression levels of the 22 genes with female-biased expression uniquely in placenta.

Supplemental Table 5. Mapping statistics for WGoxBS and in-solution target capture method.

Supplemental Table 6. Sex-specific methylation difference at the promoter regions of chromosome X genes in the placenta.

Supplemental Table 7. Programs and parameters used in this study.

Supplemental Figures

Supplemental Figure 1

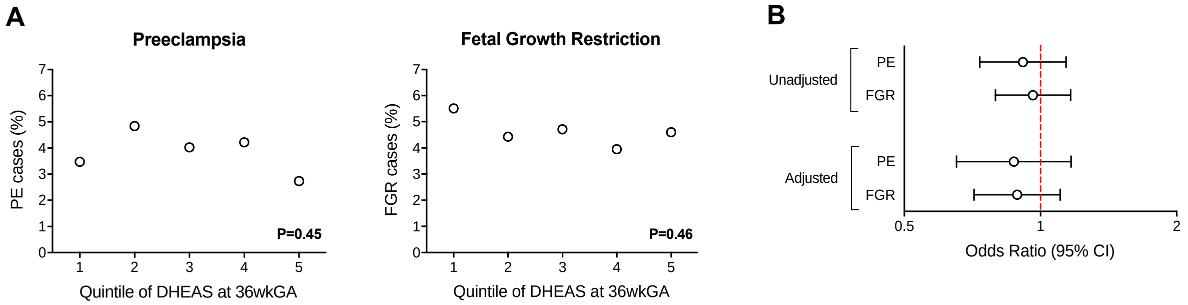

Supplemental Figure 1. Maternal serum levels of DHEAS do not change in pregnancy complications. (**A**) Proportion of cases with preeclampsia (n=134) or FGR (n=162) at term by quintile of maternal serum DHEAS at 36wkGA (two-sided logistic regression P-values are given for the linear trend between the quintile and log-odds of each outcome). (**B**) Logistic regression modelling of the association between DHEAS (as a continuous variable, ORs expressed for a 1 SD difference) at 36wkGA and the risk of preeclampsia (PE) and FGR at term both unadjusted and adjusted for sFLT1:PlGF at 36wkGA, fetal sex, maternal age, height, body mass index, ethnicity and smoking status.

Supplemental Figure 2

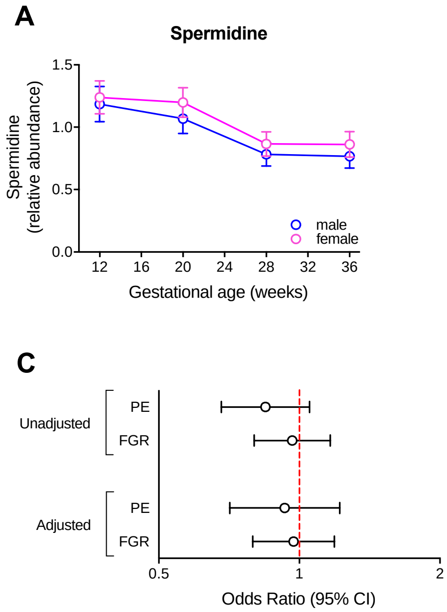

Supplemental Figure 2. Maternal serum levels of spermidine are not regulated by fetal sex and pregnancy complications. (A) Mean and 95% CI at 12, 20, 28 and 36wkGA for spermidine comparing normal pregnancies with male and female fetuses. Fetal sex differences at any of the first three gestational ages (composite hypothesis) were tested using a Chi-squared test and at 36wkGA using a t test. Two-sided P-value for the male/female difference was 0.42 at 12-20-28wkGA and 0.039 at 36wkGA. Pregnancies with female fetuses were n=146 (12, 20 and 28wkGA) and n=131 (36wkGA); pregnancies with male fetuses were n=133 (12, 20 and 28wkGA) and n=128 (36wkGA). Points=means, bars=95% CI. (B) Proportion of cases with preeclampsia (n=134) or FGR (n=162) at term by quintile of maternal serum spermidine at 36wkGA (two-sided logistic regression P-values are given for the linear trend between the quintile and log-odds of each outcome). (C) Logistic regression modelling of the association between spermidine (as a continuous variable, ORs expressed for a 1 SD difference) at 36wkGA and the risk of preeclampsia (PE) and FGR at term both unadjusted and adjusted for sFLT1:PlGF at 36wkGA, fetal sex, maternal age, height, body mass index, ethnicity and smoking status.

Supplemental References

1. ACOG. Hypertension in pregnancy. Report of the American College of Obstetricians and Gynecologists' Task Force on Hypertension in Pregnancy. *Obstet Gynecol.* 2013;122(5):1122-31.

2. Sovio U, Gaccioli F, Cook E, Hund M, Charnock-Jones DS, and Smith GC. Prediction of Preeclampsia Using the Soluble fms-Like Tyrosine Kinase 1 to Placental Growth Factor Ratio: A Prospective Cohort Study of Unselected Nulliparous Women. *Hypertension.* 2017;69(4):731-8.

3. Duncan WC, Shaw JL, Burgess S, McDonald SE, Critchley HO, and Horne AW. Ectopic pregnancy as a model to identify endometrial genes and signaling pathways important in decidualization and regulated by local trophoblast. *PloS One.* 2011;6(8):e23595.

4. Lei K, Chen L, Cryar BJ, Hua R, Sooranna SR, Brosens JJ, Bennett PR, and Johnson MR. Uterine stretch and progesterone action. *J Clin Endocrinol Metab.* 2011;96(6):E1013-24.

5. Gardosi J, Mongelli M, Wilcox M, and Chang A. An adjustable fetal weight standard. *Ultrasound Obstet Gynecol.* 1995;6(3):168-74.

6. Singh AT, Dharmarajan A, Aye IL, and Keelan JA. Sphingosine-sphingosine-1-phosphate pathway regulates trophoblast differentiation and syncytialization. *Reprod Biomed Online.* 2012;24(2):224-34.
